# Brachyury, a driver of tumor invasiveness and resistance to multiple therapies, is a novel immunotherapy target

**DOI:** 10.1186/2051-1426-1-S1-P230

**Published:** 2013-11-07

**Authors:** Claudia Palena, Romaine I Fernando, Duane H Hamilton, Bruce Huang, Jeffrey Schlom

**Affiliations:** 1 Laboratory of Tumor Immunology and Biology, Center for Cancer Research, NCI, NIH, Bethesda, MD, USA

## 

The epithelial-mesenchymal transition (EMT) is a normal developmental process that allows the conversion of epithelial, polarized and stationary cells into highly motile and invasive mesenchymal cells, a phenomenon required for the normal formation of the embryo. Recent investigations have demonstrated that a similar phenotypic switch can aberrantly take place during tumor progression, allowing epithelial cancer cells to lose cell polarity, epithelial markers and cell-to-cell contacts, while simultaneously acquiring mesenchymal-associated markers, cell motility and invasiveness. The T-box transcription factor brachyury has been recently characterized as a regulator of tumor EMT. As we demonstrate here, over-expression of brachyury in human carcinoma cells promotes tumor invasiveness in vitro and tumor dissemination in vivo, while endowing tumor cells with mechanisms of resistance to multiple therapies, including chemotherapy, radiation and some small targeted therapies. Targeting of brachyury, therefore, appears an interesting approach against tumor progression, however, due to its nuclear localization and the “undruggable” character of this kind of molecules, current options to target brachyury are limited. Our laboratory has investigated whether brachyury could be a target of an immunological approach. Our data indicate that brachyury is a tumor-associated antigen; by employing a murine monoclonal Ab against brachyury we have analyzed brachyury expression in a range of normal adult tissues and various tumor types. The brachyury protein was found expressed in non-small lung and breast carcinomas, both primary and metastases, while being absent in the majority of human adult normal tissues, with exception of low levels observed in testis and selected thyroid samples. In breast cancer, moreover, we have found an inverse association between the levels of brachyury expression and clinical outcome in patients receiving tamoxifen treatment. The immunogenicity of the brachyury protein has been characterized. A 9-mer epitope of brachyury has been identified and used to efficiently expand brachyury-specific T cells from the blood of cancer patients. We present data here on the use of these T cells to lyse brachyury-positive tumors in an antigen-specific, MHC-restricted fashion. Based on these studies, a brachyury-based yeast recombinant vaccine has been developed that is currently ongoing Phase I clinical testing in patients with advanced carcinomas.

